# Low‐dose oral cadmium increases airway reactivity and lung neuronal gene expression in mice

**DOI:** 10.14814/phy2.12821

**Published:** 2016-07-12

**Authors:** Joshua D. Chandler, Cherry Wongtrakool, Sophia A. Banton, Shuzhao Li, Michael L. Orr, Dana Boyd Barr, David C. Neujahr, Roy L. Sutliff, Young‐Mi Go, Dean P. Jones

**Affiliations:** ^1^ Department of MedicineDivision of Pulmonary, Allergy and Critical Care MedicineEmory UniversityAtlantaGeorgia; ^2^ Atlanta VA Medical CenterDecaturGeorgia; ^3^ Department of Environmental HealthRollins School of Public HealthEmory UniversityAtlantaGeorgia

**Keywords:** Airway resistance, chemosensory function, metabolomics, metal toxicity, transcriptomics

## Abstract

Inhalation of cadmium (Cd) is associated with lung diseases, but less is known concerning pulmonary effects of Cd found in the diet. Cd has a decades‐long half‐life in humans and significant bioaccumulation occurs with chronic dietary intake. We exposed mice to low‐dose CdCl_2_ (10 mg/L in drinking water) for 20 weeks, which increased lung Cd to a level similar to that of nonoccupationally exposed adult humans. Cd‐treated mice had increased airway hyperresponsiveness to methacholine challenge, and gene expression array showed that Cd altered the abundance of 443 mRNA transcripts in mouse lung. In contrast to higher doses, low‐dose Cd did not elicit increased metallothionein transcripts in lung. To identify pathways most affected by Cd, gene set enrichment of transcripts was analyzed. Results showed that major inducible targets of low‐dose Cd were neuronal receptors represented by enriched olfactory, glutamatergic, cholinergic, and serotonergic gene sets. Olfactory receptors regulate chemosensory function and airway hypersensitivity, and these gene sets were the most enriched. Targeted metabolomics analysis showed that Cd treatment also increased metabolites in pathways of glutamatergic (glutamate), serotonergic (tryptophan), cholinergic (choline), and catecholaminergic (tyrosine) receptors in the lung tissue. Protein abundance measurements showed that the glutamate receptor GRIN2A was increased in mouse lung tissue. Together, these results show that in mice, oral low‐dose Cd increased lung Cd to levels comparable to humans, increased airway hyperresponsiveness and disrupted neuronal pathways regulating bronchial tone. Therefore, dietary Cd may promote or worsen airway hyperresponsiveness in multiple lung diseases including asthma.

## Introduction

Cadmium (Cd) is a toxic environmental metal with both natural and anthropogenic sources (Satarug et al. 2003; Satarug and Moore 2004). Cd has many important commercial uses and is also present in cigarette smoke. Research on Cd toxicity has largely focused on occupational exposures and smoking, with experimental studies using appropriate dosing and routes of exposure, showing a broad spectrum of Cd‐induced toxicities to many organ systems, including lung (Waalkes 2003; Klaassen et al. 2009). Importantly, declines in smoking and environmental pollution as well as environmental remediation efforts have considerably decreased mean population exposure to Cd (Tellez‐Plaza et al. 2012) and associated health risks (García‐Esquinas et al. 2014; Nawrot et al. 2015).

Despite success in decreasing Cd exposures, ongoing dispersion of Cd occurs into air, water, and soil, and remains a significant global concern due to Cd accumulation in foodstuffs (Satarug et al. 2003; Riederer et al. 2013). Plants have no excretory system to eliminate Cd, so Cd in water and soil accumulates in leafy plants and the animals that consume them (Järup 2003; Satarug et al. 2003). In individuals without occupational exposure or smoking, ingestion is the predominant route of Cd exposure (Parikh et al. 2014). About 10% of ingested Cd is absorbed via divalent metal transporter‐1, and higher absorption occurs in menstruating women due to increased activity of intestinal iron transporters, which also transport Cd (Olsson et al. 2002). The absorbed fraction is poorly eliminated with excretion of only ~0.001% of body Cd per day and a half‐life >10 years (Waalkes 2003; Satarug and Moore 2004; Suwazono et al. 2009). Consequently, blood levels of Cd significantly increase throughout adulthood (Lee and Kim 2016).

Although total Cd exposure for US children, teens, and adults has on average decreased, dietary Cd exposure was unchanged over the period of the 1999–2008 NHANES survey (Riederer et al. 2013). Estimated Cd consumption from foodstuffs by US adults (0.30–0.35 *μ*g/kg/day) (Agency for Toxic Substances and Disease Registry, 2012) roughly equals the European Food Safety Authority guidelines of maximal tolerable exposure (0.36 *μ*g/kg/day) (European Food Safety Authority, 2011) and is at least threefold higher than the minimal risk level for chronic oral exposure stated by the US Agency for Toxic Substances and Disease Registry (0.1 *μ*g/kg/day) (Agency for Toxic Substances and Disease Registry, 2012). Importantly, few studies have addressed effects of low levels of Cd exposure to determine whether these cause or adversely contribute to common diseases.

Epidemiologic evidence shows that a lifetime of exposure to environmental levels of Cd is associated with the risk of multiple organ diseases, including the lungs (Satarug and Moore 2004; Buck Louis et al. 2012; García‐Esquinas et al. 2014), and with increased risk of adverse effects even when adjusting for lead and mercury exposure (Lee and Kim 2016). Cd accumulates in the lungs of healthy adults in proportion to environmental exposure (Mari et al. 2014), and systemic Cd exposure is linked to lung cancer, bronchitis, and emphysema (Waalkes 2003; Bertin and Averbeck 2006; Hassan et al. 2014; Oh et al. 2014; Yoon et al. 2014). Furthermore, a study of environmental tobacco smoke in children with asthma showed Cd‐containing particulates could contribute to asthma (Willers et al. 2005). However, the specific contribution of dietary Cd to lung diseases is less studied.

In this study, we used a low, orally administered dose of Cd to model dietary Cd intake. We tested whether low‐dose Cd impacted airway hyperresponsiveness (AHR) in mice and used transcriptomic analysis with gene set enrichment to assess whether oral Cd contributes to pro‐asthma pathways and other lung disease processes. Cd was administered in drinking water at an amount and duration (10 mg/L CdCl_2_, 20 weeks) projected to result in lung tissue Cd burden comparable to humans (Thijssen et al. 2007). We measured lung tissue Cd by inductively coupled plasma mass spectrometry (ICP‐MS), airway resistance with methacholine challenge, and transcript abundance by gene expression array analysis with confirmation of selected gene expression by qPCR and western blotting. Cd increased lung tissue Cd burden, AHR and neuronal gene sets related to olfactory, glutamatergic, cholinergic, serotonergic, and GABAergic pathways and related metabolic precursors. Together, these data suggest that environmental Cd found in food could contribute to asthma risk.

## Methods

### Drinking water model of low‐dose Cd exposure in mice

All animal procedures were approved by the Atlanta Veterans Affairs Medical Center IACUC (protocol number V018‐03). Previous research showed that mice with Cd in drinking water up to 100 mg/L consumed similar amounts of food and water as control and maintain similar body masses (Thijssen et al. 2007). Male C57Bl6 mice aged 8 weeks were supplied by Jackson Labs and caged in groups of five with ad libitum food (Harlan Teklad 2018S) for 20 weeks. Water was given ad libitum with 0 or 10 mg/L (55 *μ*mol/L) CdCl_2_ and fresh water was prepared weekly. The assayed Cd content of mouse chow ranged from 46–87 *μ*g/kg over the course of the study, averaging 62 ± 1 *μ*g/kg. Given an estimated daily intake of 4 g food and 6 mL water for Bl6 mice (Bachmanov et al. 2002), water comprised 99.6% of total Cd exposure for 10 mg/L CdCl_2_‐exposed mice. All mice remained healthy without weight loss or poor grooming. After 20 weeks, mice were given ketamine/xylazine, and airway resistance was measured before a lethal dose was given for the isolation of lung tissue.

### Human lung tissue acquisition and processing

To determine whether Cd levels attained in mouse lung were similar to Cd in adult human lung measured using the same methods, we obtained lung tissue from five individuals from the Emory Transplant Center. Sample collection was approved by the Emory IRB protocol (IRB000006248). Excised tissues were stored on dry ice before preparation in the same manner as mouse lung tissue for Cd measurement by ICP‐MS.

### Airway resistance measurements

After ketamine/xylazine anesthesia, respiratory mechanics were measured by forced oscillation technique (Wongtrakool et al. 2012) using the flexiVent system (SCIREQ, Montreal, Canada) with tracheotomized mice (*n* = 8). Tracheas were aseptically isolated and cannulated. Mechanical ventilation (150 breaths/min, tidal volume 8 mL/kg, PEEP 2.5 cm H_2_O) was initiated after calibration. After baseline measurements were obtained, increasing doses of inhaled methacholine were administered in 5‐min intervals. Three consecutive peak values for airway resistance at each methacholine dose were selected for analysis. Coefficients of determination were >0.95 for all values analyzed.

### Inductively coupled plasma mass spectrometry (ICP‐MS)

Mouse (*n* = 8) or human (*n* = 4) lung tissue was homogenized with a Dounce homogenizer followed by brief sonication on ice. Samples consisting of 1.5 mg protein from mouse or human lung tissue were treated with nitric acid for digestion of organic material prior to measurement by ICP‐MS. ICP‐MS procedures conformed to previously stated accuracy (100 ± 10%) and precision standards (relative standard deviation < 12%). Lung tissue Cd is reported as ng per mg lung tissue protein.

### Transcriptomics

RNA was isolated from mouse lung tissue lysate (*n* = 3) with the mirVana Total RNA Isolation Kit (Life Technologies, Carlsbad, California). RNA was hybridized on Affymetrix Mouse Gene ST 2.0 exon chips following NuGEN Ovation amplification. CEL files were converted to Robust Multi‐Array (RMA) data with R package “oligo” (Bioconductor); then, differential expression of genes was analyzed with R package “limma” (Bioconductor). CEL files and RMA data are accessible at the Gene Expression Omnibus hosted by the National Center for Biotechnology Information (accession: GSE74256). Transcripts were annotated according to the Affymetrix na33.2 annotation release. Gene Set Enrichment Analysis applet (GSEA; Broad Institute, Cambridge, MA) (Subramanian et al. 2005) was used to determine the enriched gene sets from canonical pathways. Gene sets significant at FDR‐adjusted *q* < 0.05 and containing at least one differentially expressed gene in the leading edge were then analyzed.

### Quantitative reverse transcription polymerase chain reaction RT‐qPCR

Primers were designed using the NCBI Primer‐Blast tool, with exon–exon junctions included when possible. cDNA was prepared from aliquots of the same mouse lung tissue RNA used for Affymetrix array using the Advantage RT‐for‐PCR Kit (ClonTech, Mountain View, California) and diluted 25‐fold in molecular biology grade water. A quantity of 4 *μ*L was then mixed with 16 *μ*L 1.25X iScript SYBR Green reaction master mix (Bio‐Rad, Hercules, CA) and 400 nmol/L forward and reverse primers. PCR was run for 40 cycles consisting of 20 sec each at 94°C, 56°C, and 72°C. Melt curves and gel electrophoresis were used to confirm uniform product at the expected size. A logarithmic standard curve was run with each gene to calibrate PCR reaction efficiency. Primer designs are given in Table 1.

**Table 1:** Neuronal gene targets of oral low‐dose Cd in lung tissue identified by microarray and gene set enrichment

Gene ID	Name	Avg (Cd)	Avg (Control)	Fold change	*P* value
*Olfr97*	Olfactory receptor 97	3.50 ± 0.10	2.72 ± 0.19	1.7	0.008
*Olfr458*	Olfactory receptor 458	4.10 ± 0.06	3.39 ± 0.18	1.6	0.010
*Pou5f1*	POU domain, class 5, transcription factor 1	5.50 ± 0.03	4.66 ± 0.42	1.8	0.012
*Olfr1416*	Olfactory receptor 1416	3.23 ± 0.18	2.49 ± 0.17	1.7	0.012
*Grin2a*	Glutamate receptor, ionotropic, NMDA2A	4.36 ± 0.08	3.73 ± 0.18	1.5	0.013
*Chrng*	Cholinergic receptor, nicotinic, gamma polypeptide	5.36 ± 0.07	4.95 ± 0.14	1.3	0.013
*Olfr341*	Olfactory receptor 341	5.93 ± 0.22	5.03 ± 0.27	1.8	0.018
*Htr3b*	5‐Hydroxytryptamine receptor 3B	5.22 ± 0.12	4.52 ± 0.19	1.6	0.021
*Olfr317*	Olfactory receptor 317	4.86 ± 0.14	4.32 ± 0.01	1.5	0.021
*Chrna2*	Cholinergic receptor, nicotinic, alpha polypeptide 2	4.60 ± 0.08	4.09 ± 0.16	1.4	0.023
*Omp*	Olfactory marker protein	5.03 ± 0.07	4.59 ± 0.20	1.4	0.048
*Tph2*	Tryptophan hydroxylase 2	3.83 ± 0.17	3.35 ± 0.07	1.4	0.050
*Gabrb3*	Gamma‐aminobutyric acid A receptor, subunit beta 3	5.26 ± 0.09	4.85 ± 0.08	1.3	0.053

Average intensity values are taken from the RMA table and statistical error given as SEM. Only the top 5 *Olfr* genes (by lowest *P*‐values) are listed. *n* = 3.

### Western blotting

A quantity of 20 *μ*g of protein extracted from mouse lung tissue (*n* = 4) was loaded and run on SDS‐PAGE gels, electroblotted onto PVDF, and probed with antibodies specific to GRIN2A (ab124913), olfactory marker protein (ab87338), or GAPDH (ab181602) (Abcam, Cambridge, Massachusetts). Bands were visualized using an Odyssey scanner (Li‐Cor, Lincoln, Nebraska) and intensity was quantified by NIH ImageJ software.

### Targeted analysis of neurotransmitter metabolites

Aliquots (250 *μ*g protein) of mouse lung homogenate (*n* = 8) were diluted in 70 *μ*L HPLC‐grade water and extracted with 140 *μ*L of HPLC‐grade acetonitrile containing a mixture of stable isotopic standards including [^15^N]‐choline, [^13^C_5_]‐glutamate, and [^15^N]‐tyrosine (Go et al. 2013, 2014b). Samples were incubated at 4°C for 30 min and supernatant isolated by centrifugation at 16,000 *g* for 10 min at 4°C. Metabolites were analyzed in triplicate using a high‐resolution mass spectrometry (MS) platform with C18 liquid chromatography and positive electrospray ionization on a High Field QExactive MS (Thermo, Waltham, Massachusetts) set to a scan range of 85–1275 m/z (Soltow et al. 2013). Chemical features were extracted using xMSanalyzer (Uppal et al. 2013) with apLCMS (Yu et al. 2009). Identities and *μ*mol/L abundances were established by MS/MS and coelution with authentic heavy‐isotope labeled standards and/or by comparison to reference standards (Go et al. 2015a).

### Statistics

Student's *t*‐test was used to compare Cd‐treated mice versus control for mean Cd content by ICP‐MS, gene expression, and target metabolites. Two‐way analysis of variance (ANOVA) was used to test the effect of Cd on AHR with increasing methacholine dose. One‐way ANOVA with Holm–Sidak posttest was used to test Cd content of lung tissue and Cd effect on airway hyperresponsiveness. Limma (Diboun et al. 2006) was used to evaluate gene significance from the Affymetrix RMA data matrix. Gene set significance was determined within the GSEA applet.

## Results

### Accumulation of Cd in mouse lung by low‐dose Cd exposure

**Figure 1:**
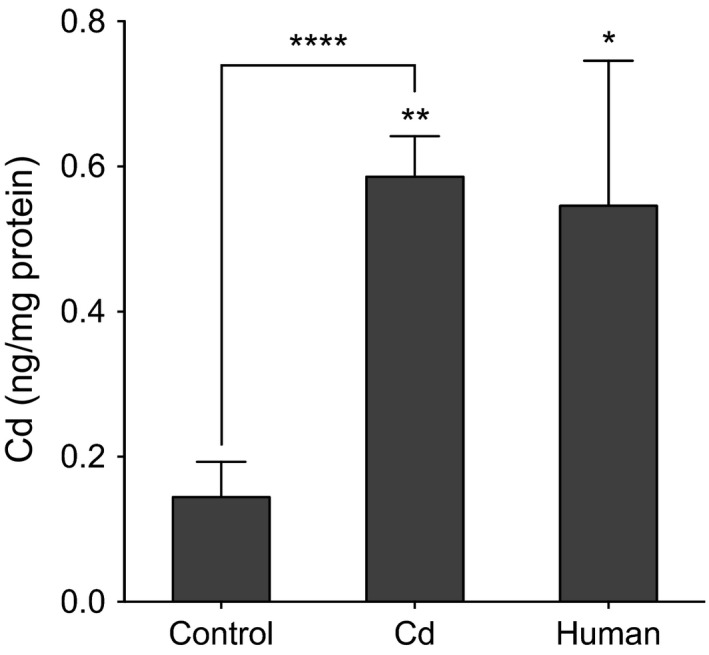
Low‐dose oral Cd exposure causes Cd lung tissue deposition comparable to human adults. Mice were exposed to 10 mg/L CdCl_2_ in drinking water (ad libitum access) for 20 weeks before sacrifice. Mice achieved comparable lung burden of Cd to that of human adults. The dashed line represents the mean of the human data set. *n* = 8 (mice) or 5 (humans). *P < 0.05, **P < 0.01 by one‐way analysis of variance (ANOVA) with Holm–Sidak posttest. ****P < 0.0001 by Student's *t* test.

We analyzed the mouse lung tissues for Cd content by ICP‐MS and found Cd concentration was substantially higher in mice treated with Cd compared with the control mice (Cd‐treated: 0.58 ± 0.06 ng Cd/mg protein, control: 0.14 ± 0.05 ng Cd/mg protein, *P* < 0.0001; Fig. 1). A small number of human lung samples (*n* = 5) were analyzed with the same methods. The results (0.54 ± 0.20 ng Cd/mg protein; Fig. 1) showed that human tissue had a similar mean value to that of the mouse model, consistent with prior data (Mari et al. 2014). Taken together, these results show that low‐dose Cd in drinking water for 20 weeks increased Cd in mouse lung tissue to levels comparable to human lungs.

### Low‐dose oral Cd increased mouse airway resistance

**Figure 2:**
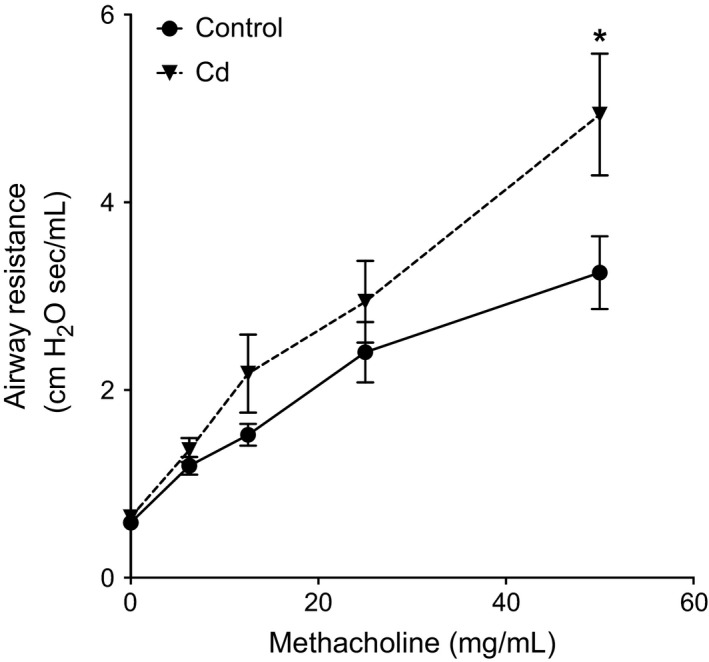
Low‐dose oral Cd exposure causes increased airway hyperresponsiveness in mice. Cd increases airway hyperresponsiveness to methacholine challenge as measured by forced oscillation technique. All measurements had a coefficient of determination of >0.95. *n* = 8. *P < 0.05 by Holm–Sidak's posttest of one‐way analysis of variance (ANOVA). Two‐way ANOVA gave concordant results (Cd 2‐way ANOVA factor *P* < 0.05).

To examine whether oral Cd exposure affected bronchial reactivity, airway resistance was assessed by the forced oscillation technique using a constant phase model (Wongtrakool et al. 2012). Mice exposed to Cd had increased airway resistance following methacholine challenge compared to unexposed control animals, maximally at 50 mg/mL of methacholine (Cd: 4.93 ± 0.65 cm H_2_O sec/mL, control: 3.25 ± 0.39; two‐way ANOVA *P* < 0.05 for Cd factor; Fig. 2). The total area under the curve of airway resistance in the Cd‐treated mice was 35% greater than control. These data show increased airway hyperresponsiveness as a result of low‐dose oral Cd exposure.

### Low‐dose Cd increased gene enrichment associated with neuronal pathways

**Figure 3:**
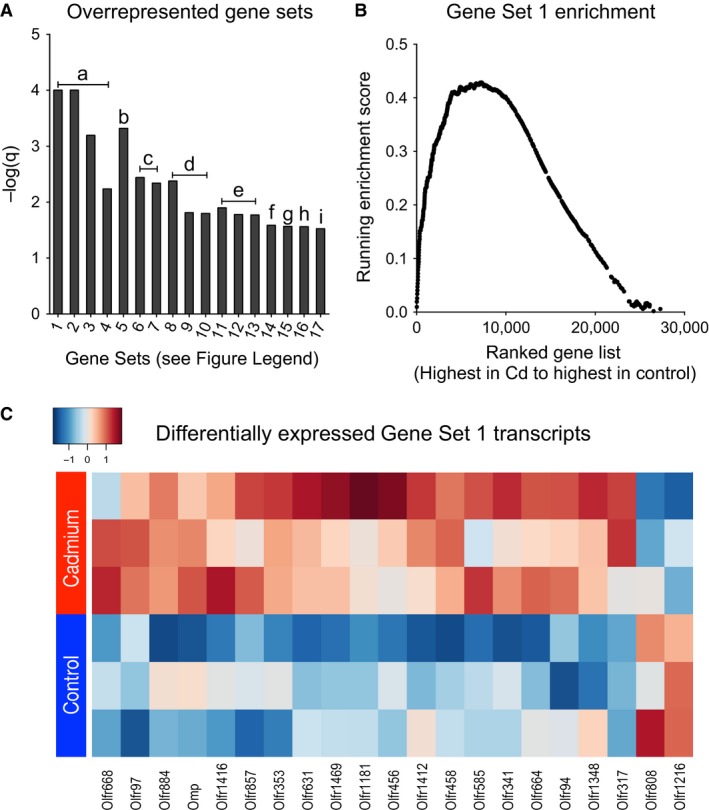
Low‐dose oral Cd increases neuronal gene set enrichment in mouse lung. (A) Gene sets significantly enriched (q < 0.05) with overrepresented gene transcripts by Cd. Numbers (*x*‐axis) denote unique gene sets and lowercase letters (in graph) denote top enriching mRNAs (see below). Gene set ID s (*x*‐axis labels): 1: Olfactory receptors. 2: Olfactory transduction. 3: GPCR downstream signaling. 4: Signaling by GPCR. 5: Neuroactive ligand receptor interaction. 6: Defensins. 7: Beta‐defensins. 8: Acetylcholine‐binding receptor and downstream events. 9: Highly calcium permeable postsynaptic nicotinic acetylcholine receptors. 10: Presynaptic nicotinic acetylcholine receptors. 11: Class A1 rhodopsin receptors. 12: Amine ligand‐binding receptors. 13: Serotonin receptors. 14: GABA A receptor activation. 15: Amine‐derived hormones. 16: GPCR ligand binding. 17: Metabolism of steroid hormones and vitamins A and D. Top enriching genes (symbols in graph): a, *Olfr97*; b, *Grin2a*; c, *Defb19*; d, *Chrna2*; e, *Htr7*; f, *Gabrb3*; g, *Tph2*; h, *Nms*; i, *Cyp27b1*. (B) Enrichment plot of Gene Set 1 from (A). Each dot is a gene set gene shown based on position in the ranked list of all genes (*x*‐axis) and its contribution to gene set enrichment (*y*‐axis). (C) Heatmap depicting autoscaled z‐scores (per column) of differentially expressed genes taken from Gene Set 1 from (A). Red means increased abundance and blue means decreased (see blue‐to‐red gradient key).

To determine whether low‐dose oral Cd exposure affected mouse lung gene expression, mRNA was isolated and analyzed using Affymetrix microarrays. Four hundred and forty‐three genes were differentially expressed at *P* < 0.05 between Cd‐treated and control groups. To test for pathway enrichment among these genes, we used Gene Set Enrichment Analysis (GSEA) to test for significant over‐ or underrepresentation of gene sets based on transcript abundance. Gene sets significantly overrepresented (FDR *q* < 0.05) following Cd exposure were predominantly neuronal in nature (Fig. 3). Olfactory receptor (*Olfr*) gene sets were most significant, including 20 differentially expressed transcripts of *Olfr* genes and olfactory marker protein (*Omp*) gene. Furthermore, glutamatergic, cholinergic, serotonergic, and GABAergic gene sets were also enriched. Differentially expressed genes within these sets included glutamatergic receptors (*Grin2a*), cholinergic receptors (*Chrna2*), serotonergic receptors (*Htr3b*), and GABAergic receptors (*Gabrb3*) (Table 1). *Pou5f1*, a gene implicated in promoter control of mouse olfactory receptor expression (Plessy et al. 2012), was also increased by Cd.

### Molecular confirmation of neuronal targets of low‐dose Cd

**Figure 4:**
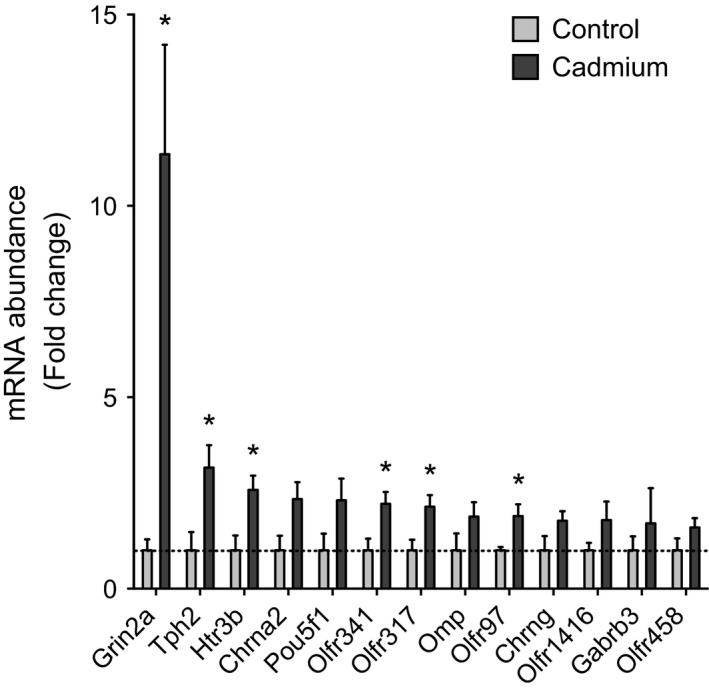
RT‐qPCR confirmation of neuronal genes increased by low‐dose oral Cd. The abundances of target neuronal genes were examined by RT‐qPCR to confirm microarray and gene set enrichment results (ordered by fold change). Primer designs are given in Table 1. Abundances were normalized to *RPS18* using the 2^−ΔΔ^ ^CT^ method. *n* = 3. *P < 0.05.

**Figure 5:**
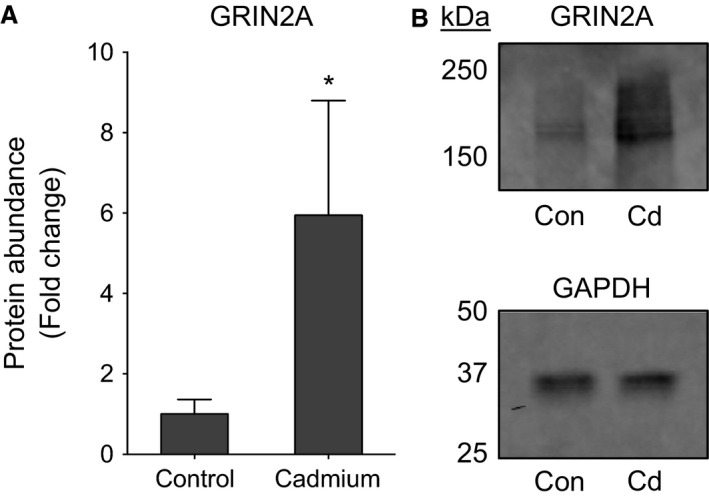
Western blotting of GRIN2A. Mouse lung tissue proteins were denatured, reduced with DTT, and separated by SDS‐PAGE on 7.5% gels. Proteins were transferred to PVDF membranes which were immunoblotted for GRIN2A (165 kDa) and GAPDH (37 kDa). Expression of GRIN2A was then normalized to GAPDH. (A) Relative quantification of lung tissue GRIN2A. (B) Membrane images of GRIN2A and GAPDH bands with molecular weight marker positions indicated to the left. *n* = 4. *P < 0.05.

To confirm microarray results of neuronal pathway enrichment, mRNA‐targeting primers were designed (Table 2) and transcript abundance was quantified by RT‐qPCR (Fig. 4). Among the genes tested, *Grin2a* had the largest fold increase (Fig. 4; 11.3‐fold of control; *P* < 0.05); genes in olfactory and serotonergic pathways were also significant, while others trended to increase by Cd. We also examined the abundance of the GRIN2A protein as the mRNA most increased by Cd in the qPCR panel and confirmed the protein level increase (Fig. 5; 5.9‐fold of control, *P* < 0.05). The quality of images for olfactory receptor protein was insufficient to determine whether this protein was increased.

**Table 2:** RT‐qPCR primers

Gene	Forward	Reverse
18S rRNA	CTG CCA TTA AGG GCG TGG G	GTG ATC ACT CGC TCC ACC TC
*Chrna2*	CAG ACT CTG GAT GGG CTG C	CTG GAA AGC AGG ATG GGA GG
*Chrng*	TGA CAG TGG GAA CGA GGA GT	AGG GAG AGC ATG GCT AGG AA
*Gabrb3*	GGG AGG AAG GCT TTT CGG CA	CGG GGT CGT TTA CGC TCT GA
*Grin2a*	TCT CCG CCT TTC CGA TTT GG	GCG TCC AAC TTC CCA GTT TT
*Htr3b*	CAC ACC TCT GAT TGG GGT CT	GGC ACA TAA GAG GCC GTT CT
*Mt2*	TCG ACC CAA TAC TCT CCG CTA	GAG CAG GAT CCA TCG GAG G
*Olfr97*	TGT TGG GAC TGT CCA GTA ACTCA	GGC CCA GCA AGT AGA GAA CAA
*Olfr317*	TGG AAG TTC TGT CCT CTC CCA T	CCG GTC AGA GAA GCC AGT AAG
*Olfr341*	TGT GCA CAC CCT TCT CTT GG	ATG GCA GGG TAA TGA CTA GTG T
*Olfr458*	CAG CAC TTT TTC GCT CTA GCC	CAG CCA CGT AGC GGT CAT AG
*Olfr1416*	CAG GCC ATT GAT ACC CGG AG	GGG GAG TAT TTC CCA ATC CCA
*Omp*	TTC TGG CGC AAG GAA GAC TC	CAG AGG CCT TTA GGT TGG CA
*Pou5f1*	GGA GGG ATG GCA TAC TGT GG	TTT CAT GTC CTG GGA CTC CTC
*Tph2*	CCC CGG AAC CAG ATA CAT GC	ATC TGA GGC TCC CAG AGA CG

Primers were designed with NCBI Primer‐Blast. All primers are listed in the 5′‐to‐3′ directionality.

### Non‐neuronal effects of low‐dose Cd

Non‐neuronal gene sets of defensins and steroid hormone synthesis were also enriched following low‐dose Cd treatment (Fig. 3A gene sets 6, 7, and 17), but none of the top genes enriching these sets were significantly different, so they were not targeted for confirmation. Additionally, many gene sets were enriched due to underrepresentation of gene transcripts, rather than by overrepresentation. These negatively enriched gene sets included cell cycle disruption, 26S proteasomal dysfunction, and cytoskeletal remodeling, consistent with Cd effects on carcinogenesis (Go et al. 2014a, 2015b).

Gene set enrichment analysis showed no significant enrichment of increased or decreased transcripts associated with metal response pathways. Because metallothionein (MT) is induced in lung tissue following acute Cd inhalation (Klaassen et al. 2009), we targeted these transcripts for confirmation. Low‐dose oral Cd did not alter the abundance of *Mt1*,* Mt3‐5*, or metal transcription factors (data not shown). Cd significantly decreased *Mt2* by microarray analysis (*P* < 0.05); qPCR showed a 3.4‐fold decrease, but this was not significant (*P* = 0.15).

### Cd altered the abundance of neurotransmitter metabolic precursors in mouse lung tissue

**Figure 6:**
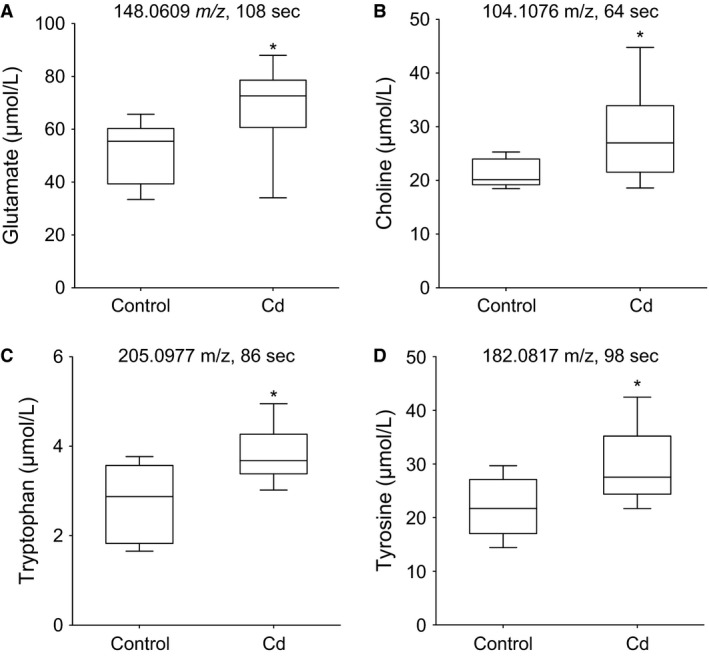
Mouse lung metabolites associated with neuronal pathways are increased by low‐dose oral Cd. Lung tissue samples were extracted using acetonitrile and analyzed by high‐resolution LC‐MS with targeted data processing for neuronal metabolites. Biological reference standards containing known concentrations of metabolites were used to estimate quantities of (A) glutamate, (B) choline, (C) tryptophan, and (D) tyrosine. *n* = 8. *P < 0.05.

To test for changes in neurotransmitter and precursor metabolite abundances, we performed metabolomics analysis on mouse lung tissue using high‐resolution mass spectrometry (Soltow et al. 2013). Multiple amino acid and amines in neuronal pathways, predominantly neurontransmitter precursors, were significantly increased by Cd (Fig. 6). These included glutamate (a co‐agonist for NDMA receptors such as GRIN2A, and a precursor of GABA), choline (an acetylcholine precursor), tryptophan (a serotonin precursor), and tyrosine (a catecholamine precursor). Furthermore, glutamate was >10‐fold more abundant than tryptophan (Fig. 6A vs. 6C), which accurately reflects the relative abundances of amino acids in the mammalian lung (Damgaard et al. 2015). Ultimately, these data show Cd‐dependent metabolic perturbations that could compound the effects of gene expression changes to lung neuronal pathways, further contributing to the observed AHR (Fig. 2) and lung disease risk.

## Discussion

Circulating Cd is predictive of airway obstruction and loss of lung function in healthy adults (Oh et al. 2014; Yoon et al. 2014) suggesting that once absorbed, Cd is a strong determinant of lung disease. This study shows that low‐dose Cd in drinking water increased mouse lung Cd to levels found in human lung (Fig. 1) and also increased AHR in the mouse model (Fig. 2). AHR (sometimes called bronchial hyperresponsiveness) is a hallmark of asthma and may also occur in COPD (Borak and Lefkowitz 2016). Clinical distinctions between the major AHR‐presenting diseases, asthma and COPD, become less clear as patients age (Slats and Taube 2016); because Cd accumulates over the human lifespan (Lee and Kim 2016), the present results suggest that dietary Cd could be generally important as a comorbidity factor in age‐associated AHR‐presenting disease.

Although the possible role, extent, and mechanism(s) of Cd in these processes are uncertain, clinical data support the potential for dietary Cd as a lung disease risk factor. Blood Cd is associated with metabolic syndrome (Lee and Kim 2016), which may cause or contribute to asthma symptoms (Serafino‐Agrusa et al. 2015). Urinary Cd measured in asthmatic children (median, 0.33 *μ*g/L) (Willers et al. 2005) is increased compared to that of NHANES children data collected the same year (mean, 0.08 *μ*g/L) (Riederer et al. 2013). Chronic respiratory symptoms are associated with urinary Cd in elderly individuals (García‐Esquinas et al. 2015), and asthma, bronchitis, and emphysema are associated with old housing which is a significant predictor of blood and urinary Cd (Shiue and Bramley 2015). However, heavy metals in urine including Cd were not associated with adult asthma in 2007–2008 NHANES data (Mendy et al. 2012) and urinary Cd was not a determinant of pro‐asthmatic respiratory symptoms in a cohort of Hong Kong preschoolers (Leung et al. 2013).

**Figure 7:**
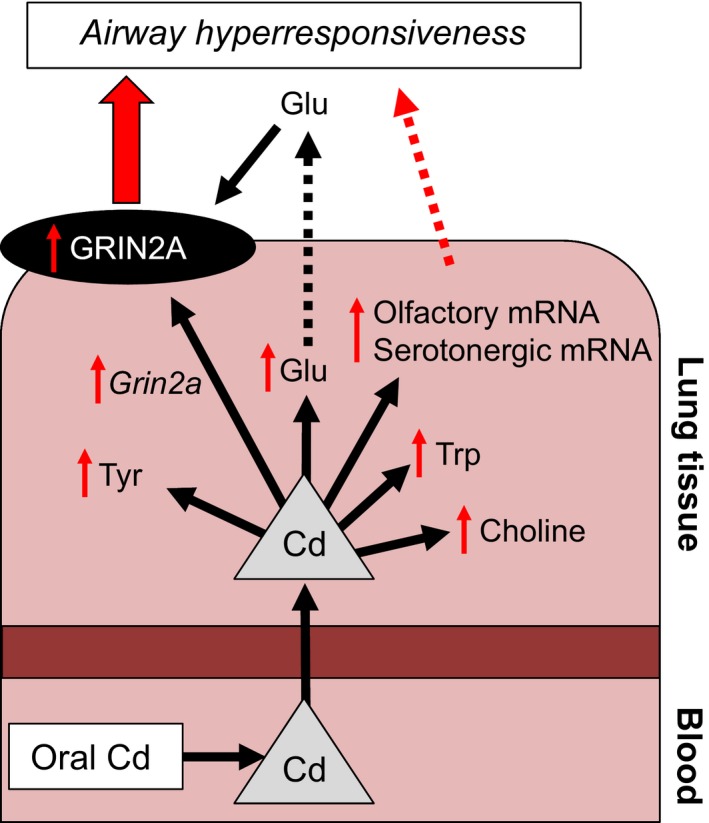
Low‐dose oral Cd increases airway hyperresponsiveness mediated by neuronal pathways. Orally absorbed Cd is transported into lung tissue and increases airway resistance by disrupting autonomic receptors and neuronal pathways. Increased NMDA receptor, ionotropic 2A (GRIN2A) protein expression, by way of increased *Grin2a* mRNA, and increased glutamate abundance coordinately contribute to increased glutamatergic activity that may drive increased airway hyperresponsiveness (AHR) in turn. Cd also increases metabolites and messenger RNA in other neuronal pathways (olfactory, serotonergic, cholinergic) which may also contribute to AHR. Therefore, orally absorbed Cd may increase AHR at the gene, protein, and metabolite levels, and this may contribute to worse symptoms in asthma, COPD, and other lung diseases.

In this study, low‐dose Cd increased neuronal receptor transcripts and metabolites suggesting contribution to lung pathophysiology, including olfactory receptors, glutamatergic receptors, glutamate, and precursor metabolites from GABAergic, serotonergic, and cholinergic pathways (Figs. 4, 5, 6; schematic summary in Fig. 7). Protein level for the glutamate‐receptor GRIN2A was also increased. Similar changes have previously been noted in control of airway resistance. Airway glutamatergic signaling was shown to control contractility in human airway smooth muscle cells and murine thin‐cut lung slices (Anaparti et al. 2015), and pulmonary neuroendocrine signaling by olfactory and serotonergic receptors was observed to regulate bronchial tone (Gu et al. 2014). Although the present design did not include Cd dose–response, we examined lung tissue transcript data from another study (JD Chandler, ML Orr, L Hao, DP Jones, and Y‐M Go, unpublished) with mice given 3.3 mg CdCl_2_ per liter of drinking water for 16 weeks. These results showed that olfactory signaling and glutamatergic pathways were among the top 20% of overrepresented gene sets. Therefore, the results are consistent with a role for Cd‐dependent induction of genes in neuronal pathways, increased GRIN2A protein, and elevation of metabolites associated with these neuronal pathways, in the mechanisms of AHR.

Asthma is epidemiologically linked with neurological disorders by hormonal and monoaminergic signaling disruption (Van Lieshout et al. 2009). Furthermore, olfactory, glutamatergic, serotonergic, and GABAergic systems may be overactive in asthma patients and others with AHR (Xiang et al. 2007; Gu et al. 2014; Anaparti et al. 2015). Effects of Cd on olfactory, serotonergic, and cholinergic pathways (Figs. 3, 4 and 6) indicate potentially broad neuroendocrine disruption in the lung. Olfactory receptors, which comprised the most overrepresented gene sets (Fig. 3), are expressed by sparsely populated pulmonary neuroendocrine cells and control airway sensitivity to volatile compounds via serotonergic and neuropeptide pathways (Gu et al. 2014). Pulmonary autonomic functions such as serotonergic signaling have previously been implicated in the control of asthma, cough, bronchitis, and pulmonary arterial hypertension (Bayer et al. 2007; Dempsie and MacLean 2008; Wang et al. 2014). Lung olfaction results in the release of metabolites to increase airway smooth muscle contractility (Dupont et al. 1999; Cazzola and Matera 2000; Gu et al. 2014).

Cd may also contribute to asthma symptoms by interacting with other factors that regulate lung disease. Iron sufficiency is a strong negative determinant of Cd burden (Olsson et al. 2002), and iron in exhaled breath is decreased in asthma (Mutti et al. 2006). Zinc is protective against Cd by the induction of metallothionein and other unspecified mechanisms (Mishima et al. 1997), and zinc can improve clinical symptoms of asthma in children such as cough, wheezing, and pulmonary function (Ghaffari et al. 2014). Selenium is decreased in the plasma of asthmatics (Fabian et al. 2013) and is a selective ionic binding partner of Cd in mammals that may help to sequester or remove Cd from the body (Arai et al. 2004). Finally, both zinc and selenium restore anti‐nitrergic effects of Cd in mice (Göçmen et al. 2000). Thus, Cd, iron, zinc, and selenium may coordinately regulate the potential contribution of Cd to AHR by direct interaction (Cd–selenium), competition for transporters (Cd–iron), or regulation of protective factors (Cd–zinc).

Many of the transcripts detected here also play roles in lung carcinogenesis. Transformed lung cells often express increased and/or polymorphic neuroendocrine receptors, including olfactory receptors (Giandomenico et al. 2013; Öberg 2015), glutamate receptors (Okayama et al. 2012; Ahn et al. 2014), and cholinergic receptors (Robles et al. 2014). Indeed, GRIN2A is increased in some lung adenocarcinomas (Okayama et al. 2012; Xin et al. 2013). Thus, while the present results demonstrate an important new pathway of AHR by dietary Cd, they also are consistent with the known lung carcinogenesis potential of Cd (Waalkes 2003).

Increased MT gene expression and protein abundance is a well‐characterized cellular response to Cd, particularly in the kidney and liver (Klaassen et al. 2009). In this study, however, Cd decreased *Mt2,* and other MT genes were not affected. This is in contrast to increased MT expression in response to acutely inhaled Cd (Hart et al. 1989; Klaassen et al. 2009). The implications of this lack of response concerning human respiratory health effects of Cd at a dose mimicking that in foodstuffs are not clear; in the worst case, the results suggest that lung may be relatively unprotected against low‐dose, chronic Cd exposure.

In conclusion, low‐dose oral Cd in mice caused lung Cd burden similar to that in adult humans, increased airway hyperresponsiveness, increased abundance of transcripts of genes controlling autonomic and chemosensory pathways, increased glutamate and other metabolites associated with these pathways, and increased glutamate‐receptor GRIN2A protein. The results suggest a new mechanism for clinical observations that Cd is associated with AHR‐associated lung diseases (Willers et al. 2005; Oh et al. 2014; Yoon et al. 2014). Given the population burden of asthma and COPD, the results emphasize an ongoing need to improve understanding of the mechanisms of low‐dose Cd toxicity, dietary sources of Cd, and possible public health impact of dietary Cd in airway hyperresponsiveness and lung disease.

## Conflicts of Interest

The authors declare no conflicts of interest.
